# Supplementary material for: Resolving the full spectrum of human genome variation using Linked-Reads
Source: Genome Res. 2019 Apr;29(4):635–45. doi: 10.1101/gr.234443.118 (PMC6442396; doi:10.1101/gr.234443.118)
Supplement: Supplemental Material [file supp_gr.234443.118_Supplemental_longranger-2.2.zip › longranger-2.2.tar.bz2!/lr2.2-public/lib/stan/cmdstan/stan/doc/api/html/grad_8hpp_source.html]

Stan: src/stan/agrad/rev/matrix/grad.hpp Source File


|  |  |
| --- | --- |
|  | Stan  2.5.0  probability, sampling & optimization |


- Main Page
- Related Pages
- Namespaces
- Classes
- Files

- File List
- File Members

All Classes Namespaces Files Functions Variables Typedefs Enumerations Enumerator Friends Macros Pages

- src
- stan
- agrad
- rev
- matrix

grad.hpp

Go to the documentation of this file.

1 #ifndef STAN\_\_AGRAD\_\_REV\_\_MATRIX\_\_GRAD\_HPP

2 #define STAN\_\_AGRAD\_\_REV\_\_MATRIX\_\_GRAD\_HPP

3

4

5 #include <stan/math/matrix/Eigen.hpp>

6 #include <stan/agrad/rev/matrix/Eigen\_NumTraits.hpp>

7 #include <stan/agrad/rev/var.hpp>

8

9 namespace stan {

10

11  namespace agrad {

12

27  void grad(var& v,

28  Eigen::Matrix<var,Eigen::Dynamic,1>& x,

29  Eigen::VectorXd& g) {

30  stan::agrad::grad(v.vi\_);

31  g.resize(x.size());

32  for (int i = 0; i < x.size(); ++i)

33  g(i) = x(i).vi\_->adj\_;

34  }

35

36  }

37 }

38

39 #endif

Eigen\_NumTraits.hpp

stan::agrad::grad

static void grad(chainable \*vi)

Compute the gradient for all variables starting from the specified root variable implementation.

**Definition:** chainable.hpp:110

stan::agrad::var::vi\_

vari \* vi\_

Pointer to the implementation of this variable.

**Definition:** var.hpp:40

var.hpp

Eigen.hpp

stan::agrad::var

Independent (input) and dependent (output) variables for gradients.

**Definition:** var.hpp:27


---

[ Stan Home Page ]

*© 2011–2014,
Stan Development Team.*
